# Supplementary material for: Light-induced switching between singlet and triplet superconducting states
Source: Nat Commun. 2024 Feb 27;15:1776. doi: 10.1038/s41467-024-45949-x (PMC10899631; doi:10.1038/s41467-024-45949-x)
Supplement: Supplementary file 1 — Supplementary Information [file 41467_2024_45949_MOESM1_ESM.pdf]

Supplementary Material: Light-induced switching between singlet and triplet superconducting states

Steven Gassner, Clara S. Weber, Martin Claassen

1 Quasiparticle excitations and pulse fluence

In Figures 4 and 5 of the main text, we report parameter regions as a function of inverse pulse width and fluence that lead to successful switching in our time-dependent Ginzburg-Landau (TDGL) simulations. In Figure 5**e**, we comment that the window of pulse widths was chosen such that fraction of fluence with frequencies in the quasiparticle continuum (defined as $\omega > 2\Delta^{(\mathrm{eq})}$, where $2\Delta^{(\mathrm{eq})}$ is the equilibrium superconducting gap) never exceeds 10%. Here we provide plots of this fluence fraction as a function of the pulse parameters. We compute this via the ratio $\int_{2\Delta^{(\mathrm{eq})}}^{\infty} |\mathbf{E}(\omega)|^2 d\omega / \int_{-\infty}^{\infty} |\mathbf{E}(\omega)|^2 d\omega$, with $\mathbf{E}(\omega) = \omega \mathbf{A}(\omega)$. The results are plotted in Figure 1.

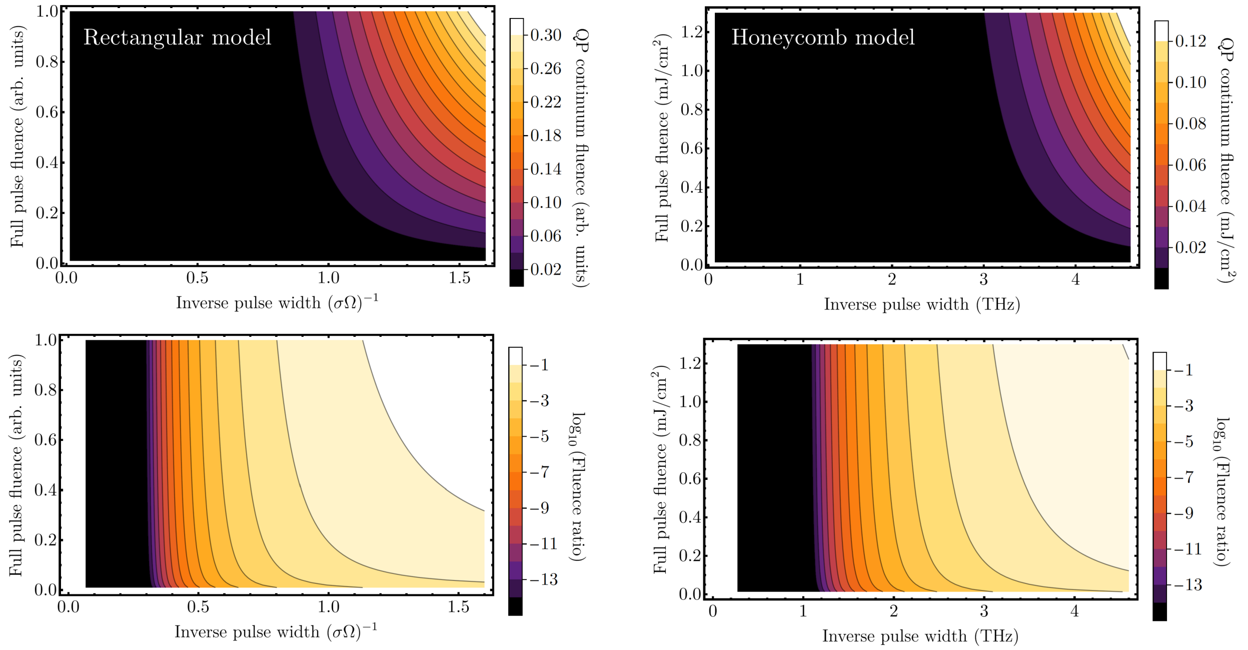

Figure 1: The amount of pulse fluence contributing to quasiparticle excitations as a function of the pulse parameters used in Figure 4 (Left, Rectangular model) and Figure 5 (Right, Honeycomb model). The upper plots calculate the fluence. The lower plots calculate the log of the ratio between the quasiparticle continuum fluence and the full fluence of the pulse.

2 Path integral derivation of the generalized Ginzburg-Landau coefficients

Here we use a path integral to derive the Ginzburg-Landau effective action presented in the main text from an arbitrary multiband Bloch Hamiltonian. We start with the partition function for the microscopic model written in terms of a path integral over the electronic Grassmann fields $\bar{\psi}$, ψ

$$\mathcal{Z} = \int \mathcal{D}[\bar{\psi}, \psi] e^{-\left(S_0[\bar{\psi},\psi] + S_{\text{int}}[\bar{\psi},\psi]\right)}, \tag{1}$$

with a free-electron action given by,

$$S_0[\bar{\psi}, \psi] = \int_0^\beta d\tau \sum_{\mathbf{k}\sigma\alpha\beta} \bar{\psi}_{\mathbf{k}\alpha\sigma} \left[\delta_{\alpha\beta}\, \partial_\tau + h^\sigma_{\alpha\beta}(\mathbf{k} + e\mathbf{A})\right] \psi_{\mathbf{k}\beta\sigma}, \tag{2}$$

and an interaction given by,

$$S_{\text{int}}[\bar{\psi}, \psi] = \int_0^\beta d\tau \, \frac{1}{L^d} \sum_{\mathbf{k}\mathbf{k}'\alpha\alpha'\beta\beta'} V_{\alpha\beta\alpha'\beta'}(\mathbf{k}, \mathbf{k}') \bar{\psi}_{\mathbf{k}-\frac{\mathbf{q}}{2},\alpha\uparrow} \bar{\psi}_{-\mathbf{k}+\frac{\mathbf{q}}{2},\beta\downarrow} \psi_{-\mathbf{k}'+\frac{\mathbf{q}}{2},\beta'\downarrow} \psi_{\mathbf{k}'-\frac{\mathbf{q}}{2},\alpha'\uparrow}. \tag{3}$$

We write the momentum indices in the "center of mass" frame of the Cooper pair and decompose the interaction function $V_{\alpha\beta\alpha'\beta'}(\mathbf{k}, \mathbf{k}')$

$$V_{\alpha\beta\alpha'\beta'}(\mathbf{k}, \mathbf{k}') = \sum_i v_i f^i_{\alpha\beta}(\mathbf{k}) \bar{f}^i_{\alpha'\beta'}(\mathbf{k}'), \tag{4}$$

where i indexes some subset of the irreducible representations (irreps) of the crystal symmetry group, and $f^i_{\alpha\beta}(\mathbf{k})$ is the momentum-space form factor associated to irrep i. We now perform a Hubbard-Stratonovich transformation to bring this action to a form that is quadratic in $\bar{\psi}$, ψ. We decouple in the Cooper channel in terms of order parameters $\bar{\Delta}_{i,\mathbf{q}}$, $\Delta_{i,-\mathbf{q}}$,

$$\begin{aligned} e^{-S_{\text{int}}[\bar{\psi},\psi]} = \int \mathcal{D}[\bar{\Delta}, \Delta] \exp\Bigg\{ &- \int_0^\beta d\tau \sum_{\mathbf{k}\,\alpha\,\beta} \sum_{i\,\mathbf{q}} \left(\bar{\Delta}_{i,\mathbf{q}} \bar{f}^i_{\beta\alpha}(\mathbf{k}) \psi_{-\mathbf{k}+\frac{\mathbf{q}}{2},\beta\downarrow} \psi_{\mathbf{k}-\frac{\mathbf{q}}{2},\alpha\uparrow} + \bar{\psi}_{\mathbf{k}-\frac{\mathbf{q}}{2},\alpha\uparrow} \bar{\psi}_{-\mathbf{k}+\frac{\mathbf{q}}{2},\beta\downarrow} \Delta_{i,-\mathbf{q}} f^i_{\alpha\beta}(\mathbf{k}) \right) \\ &+ \sum_{i\,\mathbf{q}} \bar{\Delta}_{i,\mathbf{q}} \frac{\beta L^d}{v_i} \Delta_{i,-\mathbf{q}} \Bigg\}. \end{aligned} \tag{5}$$

Here, $\mathbf{q}$ corresponds physically to the *external* momentum of a Cooper pair, in contrast to $\mathbf{k}$, which corresponds to the *internal* momentum. The action can be compactly written using Nambu fields

$$\Psi_{\mathbf{k}} \overset{\text{def}}{=} \begin{pmatrix} \psi_{\mathbf{k},1,\uparrow} \\ \vdots \\ \psi_{\mathbf{k},N,\uparrow} \\ \bar{\psi}_{-\mathbf{k},1,\downarrow} \\ \vdots \\ \bar{\psi}_{-\mathbf{k},N,\downarrow} \end{pmatrix}, \tag{6}$$

in which case the partition function takes the form,

$$\mathcal{Z} = \int \mathcal{D}[\bar{\Psi}, \Psi] \int \mathcal{D}[\bar{\Delta}, \Delta] \, \exp\left\{ - \int_0^\beta d\tau \int d^d r \left(\bar{\Psi} \hat{\mathcal{G}}^{-1} \Psi - \sum_{i\,\mathbf{q}} \bar{\Delta}_{i,\mathbf{q}} \frac{1}{v_i} \Delta_{i,-\mathbf{q}} \right) \right\}, \tag{7}$$

with the inverse Gor'kov Green's function $\hat{\mathcal{G}}^{-1}$. In Matsubara frequency representation, $\Psi(\tau) = \frac{1}{\sqrt{\beta}} \sum_{\omega_n} \Psi_{\omega_n} e^{-i\omega_n \tau}$ and therefore $\partial_\tau \to -i\omega_n$, $\int_0^\beta d\tau \to \beta \sum_{\omega_n}$. (We assume that Δ has no imaginary time dependence.) This allows writing,

$$\mathcal{Z} = \int \mathcal{D}[\bar{\Psi}, \Psi] \int \mathcal{D}[\bar{\Delta}, \Delta] \, \exp \left\{ - \sum_{\mathbf{k}\,\mathbf{k}'\,\omega_n} \bar{\Psi}_{\mathbf{k},\omega_n} \left(\hat{\mathcal{G}}^{-1} \right)^{\omega_n}_{\mathbf{k},\mathbf{k}'} \Psi_{\mathbf{k}',\omega_n} + \sum_{i\,\mathbf{q}} \bar{\Delta}_{i,\mathbf{q}} \frac{\beta L^d}{v_i} \Delta_{i,-\mathbf{q}} \right\}, \tag{8}$$

where the matrix elements $\left(\hat{\mathcal{G}}^{-1} \right)^{\omega_n}_{\mathbf{k},\mathbf{k}'}$ of the inverse Green's function operator are given by

$$\left(\hat{\mathcal{G}}^{-1} \right)^{\omega_n}_{\mathbf{k}+\frac{\mathbf{q}}{2},\mathbf{k}-\frac{\mathbf{q}}{2}} = \begin{pmatrix} \left(-i\omega_n + \hat{h}_{\mathbf{k}\uparrow} \right) \delta_{\mathbf{q},\mathbf{0}} & \sum_i \Delta_{i,-\mathbf{q}} \, \hat{f}_{\mathbf{k},i} \\ \sum_i \bar{\Delta}_{i,-\mathbf{q}} \, \hat{f}^\dagger_{\mathbf{k},i} & \left(-i\omega_n - \hat{h}^\top_{-\mathbf{k}\downarrow} \right) \delta_{\mathbf{q},\mathbf{0}} \end{pmatrix}. \tag{9}$$

Here, we use a condensed notation in which $\hat{h}_{\mathbf{k}\sigma}$ is the matrix with elements $h^\sigma_{\alpha\beta}(\mathbf{k})$, and $\hat{f}_{\mathbf{k},i}$ is the matrix with elements $f^i_{\alpha\beta}(\mathbf{k})$. Evaluating the path integral over the Grassmann fields $\bar{\Psi}$, Ψ, one obtains

$$\mathcal{Z} = \int \mathcal{D}[\bar{\Delta}, \Delta] \exp \left\{ \ln \det \hat{\mathcal{G}}^{-1} + \sum_{i\,\mathbf{q}} \bar{\Delta}_{i,\mathbf{q}} \frac{\beta L^d}{v_i} \Delta_{i,-\mathbf{q}} \right\}. \tag{10}$$

with an effective action given by

$$S_{\text{eff}}[\bar{\Delta}, \Delta] = -\text{tr} \ln \hat{\mathcal{G}}^{-1} - \sum_{i\,\mathbf{q}} \bar{\Delta}_{i,\mathbf{q}} \frac{\beta L^d}{v_i} \Delta_{i,-\mathbf{q}}. \tag{11}$$

We expand S_{eff} as a functional Taylor series around the saddle point $\Delta = 0$,

$$\begin{aligned} S_{\text{eff}}[\bar{\Delta}, \Delta] = \; & S_{\text{eff}}|_{\Delta=0} + \sum_{i\,i'\,\mathbf{q}\,\mathbf{q}'} \bar{\Delta}_{i,\mathbf{q}} \left. \frac{\delta^2 S_{\text{eff}}}{\delta \bar{\Delta}_{i,\mathbf{q}} \delta \Delta_{i',\mathbf{q}'}} \right|_{\Delta=0} \Delta_{i',\mathbf{q}'} \\ & + \frac{1}{4} \sum_{ijmn} \bar{\Delta}_{i,\mathbf{0}} \bar{\Delta}_{m,\mathbf{0}} \left. \frac{\delta^4 S_{\text{eff}}}{\delta \bar{\Delta}_{i,\mathbf{0}} \delta \Delta_{j,\mathbf{0}} \delta \bar{\Delta}_{m,\mathbf{0}} \delta \Delta_{n,\mathbf{0}}} \right|_{\Delta=0} \Delta_{j,\mathbf{0}} \Delta_{n,\mathbf{0}} \\ & + \mathcal{O}(|\Delta|^5). \end{aligned} \tag{12}$$

The first-order term vanishes by virtue of $\Delta = 0$ being a saddle point. The zeroth-order term is simply the free-electron action, which we can neglect since it just contributes an overall constant. The second-order and fourth-order terms read

$$S_{\text{eff}}[\bar{\Delta}, \Delta] = \beta L^d \sum_{i\,j\,\mathbf{q}} \bar{\Delta}_{i,\mathbf{q}} \left(-\frac{1}{v_i} \delta_{ij} + \Pi^{(2)}_{ij}(\mathbf{q}) + \frac{1}{4} \sum_{m\,n} \Pi^{(4)}_{ijmn}(\mathbf{0}) \, \delta_{\mathbf{q}\mathbf{0}} \bar{\Delta}_{m,\mathbf{0}} \Delta_{n,\mathbf{0}} \right) \Delta_{j,-\mathbf{q}}, \tag{13}$$

in terms of tensors $\Pi^{(2)}_{ij}(\mathbf{q})$ and $\Pi^{(4)}_{ijmn}(\mathbf{0})$, described in the main text.

2.1 Second-order terms

The second-order in Δ terms (including the gradient terms), are computed in terms of $\Pi^{(2)}_{ii'}(\mathbf{q})$. Comparing Eqs. (12) and (13), we find,

$$\begin{aligned}
\Pi^{(2)}_{ii'}(\mathbf{q}) &= -\frac{1}{\beta L^d}\,\frac{\delta^2}{\delta\bar{\Delta}_{i,\mathbf{q}}\delta\Delta_{i',\mathbf{q}'}}\mathrm{tr}\ln\hat{\mathcal{G}}^{-1}\Big|_{\Delta=0} \\
&= -\frac{1}{\beta L^d}\,\frac{\delta}{\delta\bar{\Delta}_{i,\mathbf{q}}}\mathrm{tr}\left(\hat{\mathcal{G}}\,\frac{\delta\hat{\mathcal{G}}^{-1}}{\delta\Delta_{i',\mathbf{q}'}}\right)\Bigg|_{\Delta=0} \\
&= \frac{1}{\beta L^d}\,\mathrm{tr}\left(\hat{\mathcal{G}}_0\frac{\delta\hat{\mathcal{G}}_0^{-1}}{\delta\bar{\Delta}_{i,\mathbf{q}}}\hat{\mathcal{G}}_0\frac{\delta\hat{\mathcal{G}}_0^{-1}}{\delta\Delta_{i',\mathbf{q}'}}\right),
\end{aligned} \tag{14}$$

where we use the shorthand $\hat{\mathcal{G}}_0 \equiv \hat{\mathcal{G}}\Big|_{\Delta=0}$. For the honeycomb lattice considered in the main text, the matrix elements of the Green's function $\hat{\mathcal{G}}_0$ are given by

$$\begin{aligned}
\left(\hat{\mathcal{G}}_0\right)^{\omega_n}_{\mathbf{k},\mathbf{k}'} &= \delta_{\mathbf{k},\mathbf{k}'}\begin{pmatrix}(-i\omega_n+\hat{h}_{\mathbf{k}\uparrow})^{-1} & 0\\ 0 & (-i\omega_n-\hat{h}^{\top}_{-\mathbf{k}\downarrow})^{-1}\end{pmatrix} \\
&\equiv \delta_{\mathbf{k},\mathbf{k}'}\begin{pmatrix}\hat{G}^{\uparrow}_{\mathbf{k}}(i\omega_n) & 0\\ 0 & \hat{G}^{\downarrow}_{\mathbf{k}}(i\omega_n)\end{pmatrix},
\end{aligned} \tag{15}$$

where we define $\hat{G}^{\uparrow}_{\mathbf{k}}$ and $\hat{G}^{\uparrow}_{\mathbf{k}}$ (non-script G's) for later convenience (and we will sometimes suppress the $i\omega_n$ dependence to declutter notation). With these definitions in order, one can carefully compute $\Pi^{(2)}_{ii'}(\mathbf{q})$ making use of the matrix elements of these operators,

$$\begin{aligned}
\Pi^{(2)}_{ii'}(\mathbf{q}) &= \frac{1}{\beta L^d}\sum_{\mathbf{k}_1\,\mathbf{k}_2\,\mathbf{k}_3\,\mathbf{k}_4\,\omega_n}\mathrm{tr}\left\{\left(\hat{\mathcal{G}}_0\right)_{\mathbf{k}_1,\mathbf{k}_2}\left(\frac{\delta\hat{\mathcal{G}}_0^{-1}}{\delta\bar{\Delta}_{i,\mathbf{q}}}\right)_{\mathbf{k}_2,\mathbf{k}_3}\left(\hat{\mathcal{G}}_0\right)_{\mathbf{k}_3,\mathbf{k}_4}\left(\frac{\delta\hat{\mathcal{G}}_0^{-1}}{\delta\Delta_{i',\mathbf{q}'}}\right)_{\mathbf{k}_4,\mathbf{k}_1}\right\} \\
&= \frac{1}{\beta L^d}\sum_{\{\mathbf{k}_j\}\,\omega_n}\mathrm{tr}\left\{\begin{pmatrix}\hat{G}^{\uparrow}_{\mathbf{k}_1} & 0\\ 0 & \hat{G}^{\downarrow}_{\mathbf{k}_1}\end{pmatrix}\delta_{\mathbf{k}_1,\mathbf{k}_2}\begin{pmatrix}0 & 0\\ \hat{f}^{\dagger}_{\frac{\mathbf{k}_2+\mathbf{k}_3}{2},i} & 0\end{pmatrix}\delta_{\mathbf{k}_3-\mathbf{k}_2,\mathbf{q}}\begin{pmatrix}\hat{G}^{\uparrow}_{\mathbf{k}_3} & 0\\ 0 & \hat{G}^{\downarrow}_{\mathbf{k}_3}\end{pmatrix}\delta_{\mathbf{k}_3,\mathbf{k}_4}\begin{pmatrix}0 & \hat{f}_{\frac{\mathbf{k}_4+\mathbf{k}_1}{2},i'}\\ 0 & 0\end{pmatrix}\delta_{\mathbf{k}_1-\mathbf{k}_4,\mathbf{q}'}\right\} \\
&= \boxed{\frac{1}{\beta L^d}\sum_{\mathbf{k}\,\omega_n}\mathrm{tr}\left\{\hat{G}^{\uparrow}_{\mathbf{k}-\frac{\mathbf{q}}{2}}(i\omega_n)\cdot\hat{f}_{\mathbf{k},i'}\cdot\hat{G}^{\downarrow}_{\mathbf{k}+\frac{\mathbf{q}}{2}}(i\omega_n)\cdot\hat{f}^{\dagger}_{\mathbf{k},i}\right\}}.
\end{aligned} \tag{16}$$

Here, $\mathbf{q}'$ must equal $-\mathbf{q}$, as imposed by the Kronecker deltas.

Performing the Matsubara sum in Eq. (16) is made easier by expressing the Green's operators $\hat{G}^{\sigma}_{\mathbf{k}}(i\omega_n)$ in terms of the Bloch eigenstates $|u^{\sigma}_{m\mathbf{k}}\rangle$,

$$\hat{G}^{\sigma}_{\mathbf{k}}(i\omega_n) = \sum_m \frac{|u^{\sigma}_{m\mathbf{k}}\rangle\langle u^{\sigma}_{m\mathbf{k}}|}{-i\omega_n+\epsilon^{\sigma}_{m\mathbf{k}}}. \tag{17}$$

This gives

$$\Pi^{(2)}_{ii'}(\mathbf{q}) = \frac{1}{\beta L^d}\sum_{mm'}\sum_{\mathbf{k}\,\omega_n}\frac{1}{(-i\omega_n+\epsilon^{\downarrow}_{m,\mathbf{k}+\frac{\mathbf{q}}{2}})(-i\omega_n+\epsilon^{\uparrow}_{m',\mathbf{k}-\frac{\mathbf{q}}{2}})}\left\langle u^{\downarrow}_{m,\mathbf{k}+\frac{\mathbf{q}}{2}}\middle|\hat{f}^{\dagger}_{\mathbf{k},i}\middle|u^{\uparrow}_{m',\mathbf{k}-\frac{\mathbf{q}}{2}}\right\rangle\left\langle u^{\uparrow}_{m',\mathbf{k}-\frac{\mathbf{q}}{2}}\middle|\hat{f}_{\mathbf{k},i'}\middle|u^{\downarrow}_{m,\mathbf{k}+\frac{\mathbf{q}}{2}}\right\rangle$$
$$= \frac{1}{\beta L^d}\sum_{mm'}\sum_{\mathbf{k}}\frac{n_{\mathrm{F}}(\epsilon^{\downarrow}_{m,\mathbf{k}+\frac{\mathbf{q}}{2}})-n_{\mathrm{F}}(\epsilon^{\uparrow}_{m',\mathbf{k}-\frac{\mathbf{q}}{2}})}{\epsilon^{\downarrow}_{m,\mathbf{k}+\frac{\mathbf{q}}{2}}-\epsilon^{\downarrow}_{m',\mathbf{k}-\frac{\mathbf{q}}{2}}}\left\langle u^{\downarrow}_{m,\mathbf{k}+\frac{\mathbf{q}}{2}}\middle|\hat{f}^{\dagger}_{\mathbf{k},i}\middle|u^{\uparrow}_{m',\mathbf{k}-\frac{\mathbf{q}}{2}}\right\rangle\left\langle u^{\uparrow}_{m',\mathbf{k}-\frac{\mathbf{q}}{2}}\middle|\hat{f}_{\mathbf{k},i'}\middle|u^{\downarrow}_{m,\mathbf{k}+\frac{\mathbf{q}}{2}}\right\rangle, \tag{18}$$

where $n_{\mathrm{F}}(x) = (1+\exp(\beta x))^{-1}$ is the Fermi-Dirac distribution function. If the system respects time-reversal symmetry, then $\hat{h}^{\uparrow}_{\mathbf{k}} = \left(\hat{h}^{\downarrow}_{-\mathbf{k}}\right)^*$, and $\Pi^{(2)}_{ii'}(\mathbf{q})$ becomes,

$$\Pi^{(2)}_{ii'}(\mathbf{q}) = \frac{1}{\beta L^d}\sum_{mm'}\sum_{\mathbf{k}}\frac{n_{\mathrm{F}}(\epsilon_{m,\mathbf{k}+\frac{\mathbf{q}}{2}})-n_{\mathrm{F}}(-\epsilon_{m',\mathbf{k}-\frac{\mathbf{q}}{2}})}{\epsilon_{m,\mathbf{k}+\frac{\mathbf{q}}{2}}+\epsilon_{m',\mathbf{k}-\frac{\mathbf{q}}{2}}}\left\langle u_{m,\mathbf{k}+\frac{\mathbf{q}}{2}}\middle|\hat{f}^{\dagger}_{\mathbf{k},i}\middle|u_{m',\mathbf{k}-\frac{\mathbf{q}}{2}}\right\rangle\left\langle u_{m',\mathbf{k}-\frac{\mathbf{q}}{2}}\middle|\hat{f}_{\mathbf{k},i'}\middle|u_{m,\mathbf{k}+\frac{\mathbf{q}}{2}}\right\rangle. \tag{19}$$

2.2 Fourth-order terms

Following steps analogous to the previous section, we find that $\Pi^{(4)}_{ijmn}(\mathbf{0})$ equals the following (assuming time-reversal symmetry of the Bloch Hamiltonian),

$$\begin{aligned}\Pi^{(4)}_{ijmn}(\mathbf{0}) \equiv & \left.\frac{\delta^4 S_{\text{eff}}}{\delta\bar{\Delta}_{i,\mathbf{0}}\delta\Delta_{j,\mathbf{0}}\delta\bar{\Delta}_{m,\mathbf{0}}\delta\Delta_{n,\mathbf{0}}}\right|_{\Delta=0} \\ = & \frac{1}{\beta L^d}\sum_{\mathbf{k}\,\omega_n}\mathrm{tr}\left\{\hat{G}^{\uparrow}_{\mathbf{k}}(i\omega_n)\cdot\hat{f}_{\mathbf{k},n}\cdot\hat{G}^{\downarrow}_{\mathbf{k}}(i\omega_n)\cdot\hat{f}^{\dagger}_{\mathbf{k},m}\cdot\hat{G}^{\uparrow}_{\mathbf{k}}(i\omega_n)\cdot\hat{f}_{\mathbf{k},j}\cdot\hat{G}^{\downarrow}_{\mathbf{k}}(i\omega_n)\cdot\hat{f}^{\dagger}_{\mathbf{k},i}\right\}.\end{aligned} \tag{20}$$

Writing this in terms of Bloch eigenstates (assuming time-reversal symmetry, for simplcity), we have,

$$\Pi^{(4)}_{i_1\ldots i_4} = \frac{1}{\beta L^d}\sum_{m_1\ldots m_4}\sum_{\mathbf{k}\,\omega_n}\frac{\langle u_{m_1\mathbf{k}}|\hat{f}_{\mathbf{k},i_1}|u_{m_2\mathbf{k}}\rangle\langle u_{m_2\mathbf{k}}|\hat{f}^{\dagger}_{\mathbf{k},i_2}|u_{m_3\mathbf{k}}\rangle\langle u_{m_3\mathbf{k}}|\hat{f}_{i_3\mathbf{k}}|u_{m_4\mathbf{k}}\rangle\langle u_{m_4\mathbf{k}}|\hat{f}^{\dagger}_{i_4\mathbf{k}}|u_{m_1\mathbf{k}}\rangle}{(-i\omega_n+\epsilon_{m_1\mathbf{k}})(+i\omega_n-\epsilon_{m_2\mathbf{k}})(-i\omega_n+\epsilon_{m_3\mathbf{k}})(+i\omega_n+\epsilon_{m_4\mathbf{k}})}. \tag{21}$$

The Matsubara sum here requires a bit more care to compute,

$$\begin{aligned}&\sum_{i\omega_n}\frac{1}{(-i\omega_n+\epsilon_{m_1\mathbf{k}})(+i\omega_n-\epsilon_{m_2\mathbf{k}})(-i\omega_n+\epsilon_{m_3\mathbf{k}})(+i\omega_n+\epsilon_{m_4\mathbf{k}})} \\ &= \frac{1}{\epsilon_{m_1\mathbf{k}}-\epsilon_{m_3\mathbf{k}}}\left[\frac{n_{\mathrm{F}}(\epsilon_{m_1\mathbf{k}})}{(\epsilon_{m_1\mathbf{k}}+\epsilon_{m_2\mathbf{k}})(\epsilon_{m_1\mathbf{k}}+\epsilon_{m_4\mathbf{k}})}-\frac{n_{\mathrm{F}}(\epsilon_{m_3\mathbf{k}})}{(\epsilon_{m_3\mathbf{k}}+\epsilon_{m_2\mathbf{k}})(\epsilon_{m_3\mathbf{k}}+\epsilon_{m_4\mathbf{k}})}\right] \\ &- \frac{1}{\epsilon_{m_2\mathbf{k}}-\epsilon_{m_4\mathbf{k}}}\left[\frac{n_{\mathrm{F}}(-\epsilon_{m_2\mathbf{k}})}{(\epsilon_{m_2\mathbf{k}}+\epsilon_{m_1\mathbf{k}})(\epsilon_{m_2\mathbf{k}}+\epsilon_{m_3\mathbf{k}})}-\frac{n_{\mathrm{F}}(-\epsilon_{m_4\mathbf{k}})}{(\epsilon_{m_4\mathbf{k}}+\epsilon_{m_1\mathbf{k}})(\epsilon_{m_4\mathbf{k}}+\epsilon_{m_3\mathbf{k}})}\right] \\ &\equiv Q(\epsilon_{m_1\mathbf{k}},\epsilon_{m_2\mathbf{k}},\epsilon_{m_3\mathbf{k}},\epsilon_{m_4\mathbf{k}})+Q(-\epsilon_{m_2\mathbf{k}},-\epsilon_{m_1\mathbf{k}},-\epsilon_{m_4\mathbf{k}},-\epsilon_{m_3\mathbf{k}}),\end{aligned} \tag{22}$$

where for convenience we define a function $Q(\epsilon_1,\epsilon_2,\epsilon_3,\epsilon_4)$,

$$Q(\epsilon_1,\epsilon_2,\epsilon_3,\epsilon_4) \stackrel{\text{def}}{=} \frac{1}{\epsilon_1-\epsilon_3}\left[\frac{n_{\mathrm{F}}(\epsilon_1)}{(\epsilon_1+\epsilon_2)(\epsilon_1+\epsilon_4)}-\frac{n_{\mathrm{F}}(\epsilon_3)}{(\epsilon_3+\epsilon_2)(\epsilon_3+\epsilon_4)}\right]. \tag{23}$$

Q has a well-defined limit as ϵ_1 approaches ϵ_3

$$Q_0(\epsilon_2,\epsilon_3,\epsilon_4) \stackrel{\text{def}}{=} \lim_{\epsilon_1\to\epsilon_3} Q(\epsilon_1,\epsilon_2,\epsilon_3,\epsilon_4) = \frac{n_{\mathrm{F}}'(\epsilon_3)}{(\epsilon_3+\epsilon_2)(\epsilon_3+\epsilon_4)} - n_{\mathrm{F}}(\epsilon_3)\left[\frac{1}{(\epsilon_3+\epsilon_2)(\epsilon_3+\epsilon_4)^2} + \frac{1}{(\epsilon_3+\epsilon_2)^2(\epsilon_3+\epsilon_4)}\right], \tag{24}$$

where $n_{\mathrm{F}}'(x)$ is the derivative of the Fermi-Dirac distribution function. The combined formal expression for $\Pi^{(4)}_{i_1\ldots i_4}$ reads

$$\Pi^{(4)}_{i_1\ldots i_4} = \frac{1}{\beta L^d}\sum_{m_1\ldots m_4}\sum_{\mathbf{k}}\left[Q(\epsilon_{m_1\mathbf{k}},\epsilon_{m_2\mathbf{k}},\epsilon_{m_3\mathbf{k}},\epsilon_{m_4\mathbf{k}}) + Q(-\epsilon_{m_2\mathbf{k}},-\epsilon_{m_1\mathbf{k}},-\epsilon_{m_4\mathbf{k}},-\epsilon_{m_3\mathbf{k}})\right] \times \langle u_{m_1\mathbf{k}}|\hat{f}_{\mathbf{k},i_1}|u_{m_2\mathbf{k}}\rangle\langle u_{m_2\mathbf{k}}|\hat{f}^\dagger_{\mathbf{k},i_2}|u_{m_3\mathbf{k}}\rangle\langle u_{m_3\mathbf{k}}|\hat{f}_{i_3\mathbf{k}}|u_{m_4\mathbf{k}}\rangle\langle u_{m_4\mathbf{k}}|\hat{f}^\dagger_{i_4\mathbf{k}}|u_{m_1\mathbf{k}}\rangle, \tag{25}$$

where it is understood that the proper limit Q_0 must be explicitly implemented whenever the first and third argument of Q are equal.
